# Intrinsic brain functional connectivity patterns in alcohol use disorder

**DOI:** 10.1093/braincomms/fcac290

**Published:** 2022-11-04

**Authors:** Nasim Maleki, Kayle S Sawyer, Sarah Levy, Gordon J Harris, Marlene Oscar-Berman

**Affiliations:** Department of Psychiatry, Massachusetts General Hospital, Harvard Medical School, Boston, MA 02129, USA; Psychology Research Service, VA Healthcare System, Jamaica Plain Campus, Boston, MA 02130, USA; Psychology Research Service, VA Healthcare System, Jamaica Plain Campus, Boston, MA 02130, USA; Department of Anatomy & Neurobiology, Boston University School of Medicine, Boston, MA 02118, USA; Department of Radiology, Massachusetts General Hospital, Harvard Medical School, Boston, MA 02129, USA; Sawyer Scientific, LLC, Boston, MA 02130, USA; Department of Neurology, Icahn School of Medicine at Mount Sinai, New York, NY 10029, USA; Department of Radiology, Massachusetts General Hospital, Harvard Medical School, Boston, MA 02129, USA; Psychology Research Service, VA Healthcare System, Jamaica Plain Campus, Boston, MA 02130, USA; Department of Anatomy & Neurobiology, Boston University School of Medicine, Boston, MA 02118, USA; Department of Radiology, Massachusetts General Hospital, Harvard Medical School, Boston, MA 02129, USA

**Keywords:** alcohol use disorder, fMRI, functional connectivity

## Abstract

Alcohol use disorder is associated with damaging effects to the brain. This study aimed to examine differences in static and dynamic intrinsic functional connectivity patterns in individuals with a history of alcohol use disorder in comparison to those with no history of alcohol abuse. A total of 55 participants consisting of 23 patients and 32 control individuals underwent neuropsychological assessments and resting-state functional magnetic resonance imaging on a 3 Tesla MRI scanner. Differences in functional connectivity between the two groups were determined using static and dynamic independent component analysis. Differences in static functional connectivity between the two groups were identified in the default mode network, attention network, frontoparietal network, frontal cortical network and cerebellar network. Furthermore, the analyses revealed specific differences in the dynamic temporal characteristics of functional connectivity between the two groups of participants, in a cluster involving key regions in reward, sensorimotor and frontal cortical functional networks, with some connections correlating with the length of sobriety and some others with the severity of drinking. The findings altogether suggest dysregulation in the intrinsic connectivity of cortico-basal ganglia-thalamo-cortical loops that may reflect persistent alcohol use disorder-related network abnormalities, compensatory recovery-related processes whereby additional neural resources are recruited to achieve normal levels of performance, or a predisposition toward developing alcohol use disorder.

## Teaser

This study examined static and dynamic intrinsic functional connectivity patterns of the brain associated with alcohol use disorder. The findings altogether reflect dysregulation in the temporal dynamics of the intrinsic connectivity of the cortico-basal ganglia-thalamo-cortical loops involving brain regions from reward, sensorimotor and frontal cortical networks.

## Graphical Abstract

**Graphical abstract:**
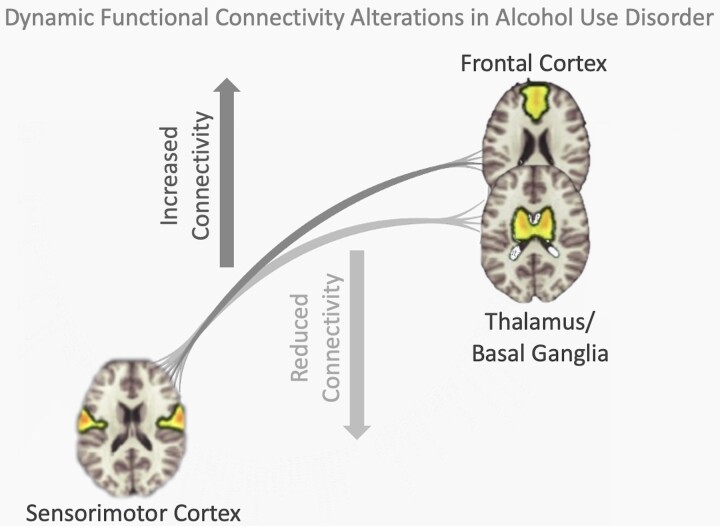

## Introduction

Chronic and excessive alcohol consumption is a significant public health problem in the USA and among the leading causes of onset of cognitive impairment and dementia. According to the 2019 National Survey on Drug Use and Health,^1^ 14.5 million individuals older than 12 years of age (9 million men and 5.5 million women) reported having alcohol use disorder (AUD).

Researchers have identified behaviours that perpetuate AUD, but the underlying neural mechanisms are less established. Clinical studies of alcohol-related cognitive deficits in individuals with chronic alcoholism have highlighted neuropsychological deficits in tasks requiring sensory input, as well as tasks that place demands on cognitive flexibility, visuospatial ability, learning and memory functions, and psychomotor speed,^2^ with relative sparing of verbal skills.^3^ While the neural substrates and mechanisms are less established,^4^ AUD is associated with widespread abnormalities in the brain, as evidenced by neuroimaging and post-mortem studies.^5–7^

These abnormalities are thought to underlie neuropsychological impairments spanning cognitive, affective and behavioural domains, as well as brain changes at multiple structural, functional and metabolic levels.^8–10^ Microstructural damage to white matter, atrophy in multiple cerebral and cerebellar regions, and ventricular enlargement, as well as functional changes in reward circuitry have all been documented.^11–15^ Correlations between these brain changes and drinking-related measures such as the duration of AUD, the amount of alcohol consumed and the duration of abstinence, also have been reported.^13,16–18^

A variety of neuroimaging techniques have been used in studying neural substrates of AUD.^6,13^ Among these techniques, functional magnetic resonance imaging (fMRI) has been employed for examining brain function and dysfunction in AUD. The most widely used fMRI method is based on the measurement of hemodynamic changes in blood oxygenation level dependent (BOLD) signals in response to specific stimuli or tasks. These vascular signals are coupled to brain metabolism and provide an indirect measure of neuronal activity.^19^ When there is no explicit stimulation or task involved, the brain is considered to be in a *resting state*, although the brain is not in fact ‘resting’. Rather, the brain remains active even without specific external stimulation, and activity is internally directed. Historically, resting-state research and analysis methods have expanded neuroscientific perspectives on brain functional architecture.^20^ The approach also can be employed to provide insights into brain abnormalities associated with neurobehavioural disorders such as AUD.^21^ Resting-state fMRI scans (rs-fMRI) have traditionally been examined using intrinsic functional connectivity analyses (fc-fMRI), which calculate co-activation patterns among different regions of the brain (nodes), revealing networks of interconnected regions. These spontaneous BOLD fluctuations tend to co-activate among anatomically distinct but functionally related networks, often referred to as intrinsic connectivity (IC) networks. For example, sometimes motor regions of the cerebellum are spontaneously more active at the same time as primary motor cortex, even if a participant is engaged in a visual task or no explicit task at all.^22^ Compared with task-related fMRI analyses, the fc-fMRI technique has been used infrequently in the context of AUD. Moreover, few studies have employed a data-driven approach examining whole-brain intrinsic functional connectivity patterns.^23^ Instead, studies either have focused on specific networks among subsets of brain regions,^24^ or have relied on regional/local neural connectivity patterns to inform regions for which functional connectivity patterns get examined.^21^ The most commonly reported findings from studies in AUD point to alterations in within-network or between-network functional connectivity patterns of reward circuitry, the default mode network or the salience network.^24–26^

Because connectivity patterns in the brain are not stationary across time, assessing dynamic functional connectivity offers additional insights into the brain’s functional organization rather than traditionally assessing functional connectivity patterns that are stationary in nature and represent static functional connectivity. While static connectivity can be thought of as a reflection of level of connection within or between specialized networks in the brain, dynamic functional connectivity can be thought of as a reflection of time-dependent and dynamic characteristics. In this study, we employed rs-fMRI in a data-driven approach to examine whole-brain intrinsic brain functional connectivity patterns in abstinent AUD individuals in comparison to a control group (CTRL) with no history of alcohol misuse. We examined differences between the groups in static and dynamic functional connectivity patterns, using independent component analysis (ICA) and dynamic independent component analysis (dynamic ICA). Thus, we looked at the functional organization of the brain at multiple levels. We sought not only to confirm salient patterns observed in prior research, but also to extend those and provide new insights into the underlying neurocircuitry of AUD at a network level.

## Materials and methods

### Participants

The Institutional Review Boards of the Massachusetts General Hospital, Boston University School of Medicine and the Boston Department of Veteran Affairs Healthcare facility approved the research. Participants were recruited between March 2010 and December 2013 through advertisements online and in newspapers in the Boston area, as well as through the participating institutions. Although written informed consent was obtained from 60 participants (26 AUD), five individuals (one AUD man and two women; two CTRL men) were excluded from analyses due to excessive motion adversely affecting fMRI signals during data preprocessing. The neuroimaging studies were performed at the Martinos Center for Biomedical Imaging at Massachusetts General Hospital, and participants were reimbursed for their time and effort.

Selection procedures included an initial telephone interview to determine age, education, health history and history of alcohol and drug use. Eligible individuals were invited to the laboratory for further screening and evaluations. Characteristics and evaluation results of the AUD and CTRL study cohorts are summarized in Tables 1 and 2. The individuals in the AUD group met diagnostic and statistical manual of mental disorders, fourth edition (DSM-IV) criteria for lifetime alcohol abuse or dependence for a minimum duration of 5 years and were abstinent for at least four weeks prior to enrolment to preclude acute effects of ethanol and withdrawal effects.^27^ All participants underwent screening for medical history, as well as alcohol and drug use. They completed a version of the Alcohol Use Disorders Identification Test (AUDIT) modified to specify past tense, a questionnaire developed by the World Health Organization.^28^ They also performed a computer-assisted, shortened version of the Diagnostic Interview Schedule^29^ that allows identifying lifetime psychiatric diagnoses following the DSM-IV criteria.^30^

**Table 1:** Participant characteristics

	AUD (*n* = 23) *Mean (SD)*	CTRL (*n* = 32) *Mean (SD)*
Age (years)	49.6 (9.7)	48.6 (14.7)
Education (years)*	14.1 (2.0)	15.6 (2.7)
Body mass index*	27.8 (5.0)	24.7 (6.5)
AUDIT score**	27.5 (5.4)	2.9 (2.2)
Daily drinks**	10.9 (8.7)	0.3 (0.3)
Duration of heavy drinking (years)**	14.9 (6.5)	0.3 (1.4)
Length of sobriety (years)*	5.2 (7.2)	0.9 (4.4)
	*Percentage (%)*	*Percentage (%)*
Gender (%men, %women)	43.0%, 57.0%	50.0%, 50.0%
Race (%white, %black, %other)	73.9%, 21.7%, 4.3%	84.4%, 12.5%, 3.1%
Neighbourhood Wealth (% average)	82.6%	90.6%
AUD family history (% first degree relative)**	87.0%	31.3%

* Indicates *P* < 0.05, **Indicates *P* < 0.001.

**Table 2:** Scores on neuropsychiatric and behavioural measures

	AUD (*n* = 23) *Mean (SD)*	CTRL (*n* = 32) *Mean (SD)*
Anxiety*	52.2 (18.0)	43.9 (7.3)
Depression	54.6 (19.7)	46.5 (10.8)
Dysphoria*	51.7 (17.9)	42.9 (7.5)
Hostility	48.4 (10.8)	45.1 (6.1)
Positive affect*	58.2 (11.6)	63.6 (7.0)
Positive affect sensation seeking*	56.0 (10.7)	61.2 (5.8)
Sensation seeking	49.5 (7.9)	52.0 (9.5)

Assessments were based on evaluations using the multiple affect adjective check list. *Indicates *P* < 0.05.

### Inclusion and exclusion criteria

The participants were right-handed and spoke English as one of their first languages. Individuals were excluded if they had one of the following: Korsakoff’s syndrome, HIV, cirrhosis, major head injury with loss of consciousness over 30 min, stroke, epilepsy or seizures unrelated to alcoholism, schizophrenia, electroconvulsive therapy, history of illicit drug use once per week or more within the past 4 years, or if they failed screening for MRI scanning.

### Neuropsychological and behavioural assessments

Upon enrolment, all participants were further assessed using a battery of neuropsychological and behavioural assessment tools. The Multiple Affect Adjective Check List^31^ was employed to evaluate positive and negative affective states and assessing depression, anxiety and sensation seeking. Estimates of alcohol use history were based on structured interviews regarding the participants’ drinking patterns, and included: daily drinks approximating the daily ounces of ethanol consumed, estimated based on the amount and type of alcohol consumed over the last six months for CTRL individuals, or over the six months preceding cessation of drinking for AUD individuals; Length of sobriety (LOS) estimated as the length of time between the date of last drink and the date of the MRI scan; and duration of heavy drinking indicating the duration of drinking 21 or more drinks per week (one drink corresponding to 355 ml beer, 148 ml wine or 44 ml hard liquor). Independent *t*-tests were performed to examine group differences in neuropsychological and behavioural measures.

### Neuroimaging data acquisition

Imaging was performed using a 3 Tesla Siemens Magnetom Trio MRI scanner and a 32-channel phased-array head coil. The resting-state functional scan was performed using a single-shot echo-planar sequence with the following parameters: repetition time = 5500 ms; echo time = 32 ms; flip angle = 90°; field of view = 256 × 256 mm; 75 interleaved axial slices with in-plane resolution 2 × 2 mm; slice thickness = 2 mm (no skip); acquisition time: 10 min, 5 s. In addition, we acquired anatomical T1-weighted images using high-resolution multi-echo magnetization prepared rapid gradient echo sequence that was used during preprocessing for registration of functional images.

### Neuroimaging data processing

Resting-state fMRI data were processed using the CONN toolbox version 20b. The preprocessing pipeline included: functional realignment and unwarping; slice-timing correction; outlier identification using ART; segmentation into grey matter, white matter and CSF; co-registration to T1 images; performing spatial normalization to the Montreal Neurological Institute space; and smoothing with a 5-mm full-width-at-half-maximum Gaussian kernel. Furthermore, fMRI signal timeseries were linearly detrended and band-pass filtered with cut-off frequencies 0.008–0.09 Hz. Structural T1 images were skull-stripped, segmented and normalized to the Montreal Neurological Institute template as well yielding normalized structural volumes. Differences in the functional connectivity between the AUD and CTRL groups were determined at two levels, while controlling for the effects of age and sex, using: (i) ICA and (ii) dynamic ICA.

Independent component analysis was performed to decompose and organize brain regions into spatially independent components representing voxels showing correlated patterns of BOLD signal time course. The group-ICA approach used in this study identified major brain networks across both groups by estimating the number of components from the aggregate dataset of both groups, which reflect the group-invariant resting-state networks. In other words, all subjects were entered into an ICA analysis and one set of components was estimated. This approach has the advantage of allowing components in different subjects to be ordered in the same way and producing a single set of group components for interpretation. CompCor algorithm^32,33^ was used to control for confounding subject-level correlations due to physiological noise resulting from cardiac pulsations and respiratory modulations, and confounding effects of white matter and CSF signals. Estimated head motion and scrubbing-related parameters were used as additional covariates. We used the default setting of the CONN functional connectivity toolbox of 40 independent components. Functional networks (and noise components as well) were identified with visual inspection and guided by comparison to functional networks consistently reported by other studies. Furthermore, we confirmed the validity of the identified networks by estimating the correlation between each group-level spatial map and CONN’s default template networks.

Dynamic ICA was performed to examine temporal characteristics of brain functional connectivity across time using an iterative dual regression on concatenated BOLD timeseries data across all participants, followed by ICA analyses (while controlling for confounding variables), and generalized psycho-physiological Interaction (gPPI) back-projection. Since differences in the resting-state component between the groups might be caused by changes in connectivity between specific regions in that component, this approach is essentially comprised of gPPI interaction terms between component timeseries that serve as psychological factors, and ROI BOLD timeseries that serve as physiological factors. This technique allows identifying clusters of connections that show similar patterns of temporal functional variation in their functional connectivity over time. The number of factors was set to 20, and the smoothing kernel was set to 30 s per the default setting of CONN. A total of 164 ROIs were used, of which 132 were from the Functional Magnetic Resonance Imaging of the Brain Software Library Harvard-Oxford atlases (91 cortical ROIs and 15 subcortical ROIs)^34–37^ and 26 cerebellar ROIs from the Automated Anatomical Labeling atlas.^38^ The remaining 32 ROIs were from atlases of networks provided in the CONN toolbox that included the default mode network, as well as salience, sensorimotor, dorsal attention, frontoparietal, language, visual and cerebellar networks.

Additional analyses were performed to better integrate the ICA and dynamic-ICA findings and understand the functional relevance of observed differences between the two groups. First, we examined the temporal properties of the BOLD signal timeseries associated with each component in the ICA analysis to identify any potential links to the dynamic ICA findings. Specifically, the temporal variability of the BOLD signal timeseries was estimated for each subject by calculating the standard deviation of each network BOLD timeseries, and the differences between the two groups were determined while accounting for age and gender. Temporal variability in the BOLD timeseries of a component determined through ICA is reflective of fluctuating communication among brain regions in that component over time. We also examined the correlation between the AUD-related indices and the connectivity strength of the regions identified through dynamic-ICA to establish the functional relevance of the findings.

### Statistical analysis

All statistical group-level comparisons were implemented in the CONN toolbox. In all analyses, we examined differences between AUD and CTRL groups while controlling for age and sex differences using a general linear model. In all comparisons, the uncorrected voxel-level statistical significance threshold was set at *P* < 0.001. In ICA comparisons, cluster-level false discovery rate (FDR) corrected threshold was set at *P* < 0.05 based on Gaussian random field theory. In dynamic-ICA comparisons, cluster-based inference was based on cluster-level *p*-FDR-corrected threshold of *P* < 0.05 and using a multi-variate pattern analysis (MVPA) omnibus test. The uncorrected region to region connection threshold in this analysis was also set at *P* < 0.05. Within the cluster/network of regions identified in the dynamic ICA, correlation between the strengths of the identified connections between any two regions and AUD indices (LOS, duration of heavy drinking and daily drinks) was determined in SPSS using bivariate correlation analysis with a significance threshold set at *P* < 0.05. This analysis was limited to AUD individuals with the goal of further examining the link between the indices specific to AUD and observed connectivity pattern differences between AUD and CTRL groups.

### Data availability

Data that support the findings of this study are available on request from the corresponding author. The data are not publicly available because they contain information that could compromise the privacy of research participants.

## Results

### Participant characteristics

A total of 60 participants met the inclusion and exclusion criteria of the study and underwent rs-fMRI scanning, but the scans of five were unusable because of motion artefacts. The remaining 55 participants included 23 AUD (10 men) and 32 CTRL (16 men) individuals. As can be seen in Table 1, differences between the two groups in age, level of education and body mass index were insubstantial and the racial distributions of both groups were similar to the demographics of Massachusetts, where the majority of participants identified as White (AUD: 73.9%, CTRL: 84.4%). Current neighbourhood wealth levels, as a measure of socio-economic status, also were comparable between the two groups, with the percentage of those living in average or above average wealth neighbourhoods slightly higher in the CTRL group (AUD: 82.6%, CTRL: 90.6%).

### Alcohol use

As expected, the average AUDIT score was significantly (*P* < 0.001) higher in the AUD group than in the CTRL group (AUD: 27.5 ± 5.4, CTRL: 2.9 ± 2.2), as were the years of heavy drinking and the number of Daily Drinks (*P* < 0.001). Because many of the CTRL participants drank alcohol occasionally, we calculated the length of sobriety in all participants as the time between their last drink and time of scan. Group differences were significant (*P <* 0.008). Finally, having first-degree family history of AUD was significantly higher (*P* < 0.001) in the AUD group (AUD: 87%, CTRL: 31.3%). Details are reported in Table 1.

### Neuropsychological findings

Evaluation of affective states using the Multiple Affect Adjective Check List revealed significantly higher scores for anxiety (*P <* 0.05) and dysphoria (*P <* 0.05) and lower scores for positive affect (*P <* 0.05) and positive affect sensation seeking (*P* < 0.05) in the AUD group compared with the CTRL group (see Table 2).

### Neuroimaging findings

**Figure 1 Common functional connectivity networks.**
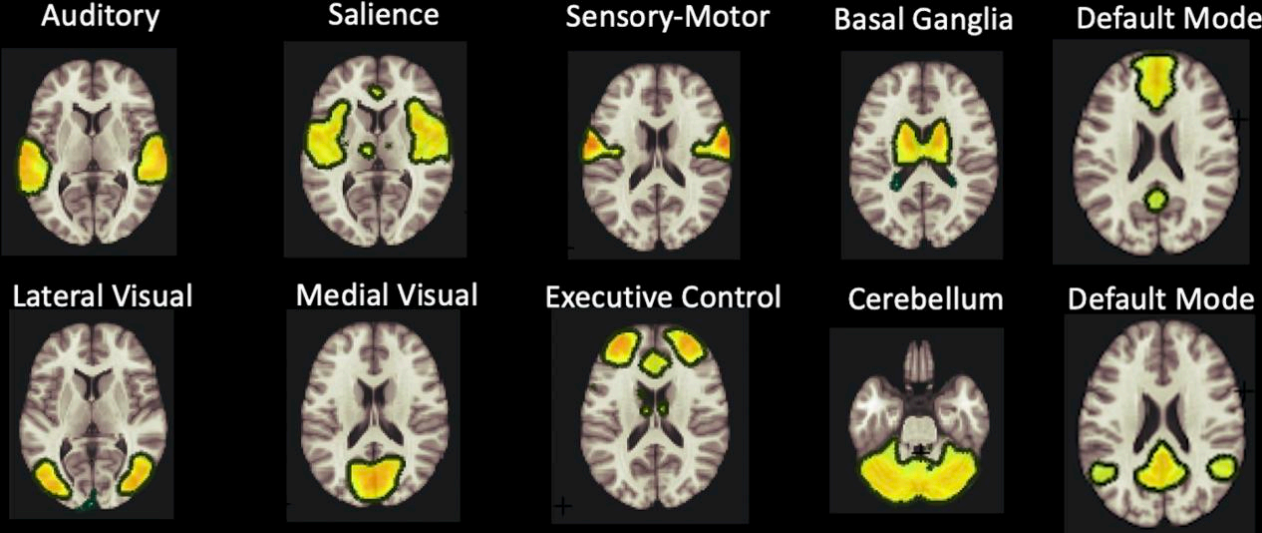
Example of spatial representation maps is shown for ten of the major independent components identified at a group level in the entire sample (the AUD and CTRL groups) using ICA. The maps represent *z*-statistic scores thresholded at *z* = 2 (range = 0–11).

**Figure 2 Differences in functional connectivity of the default mode network.**
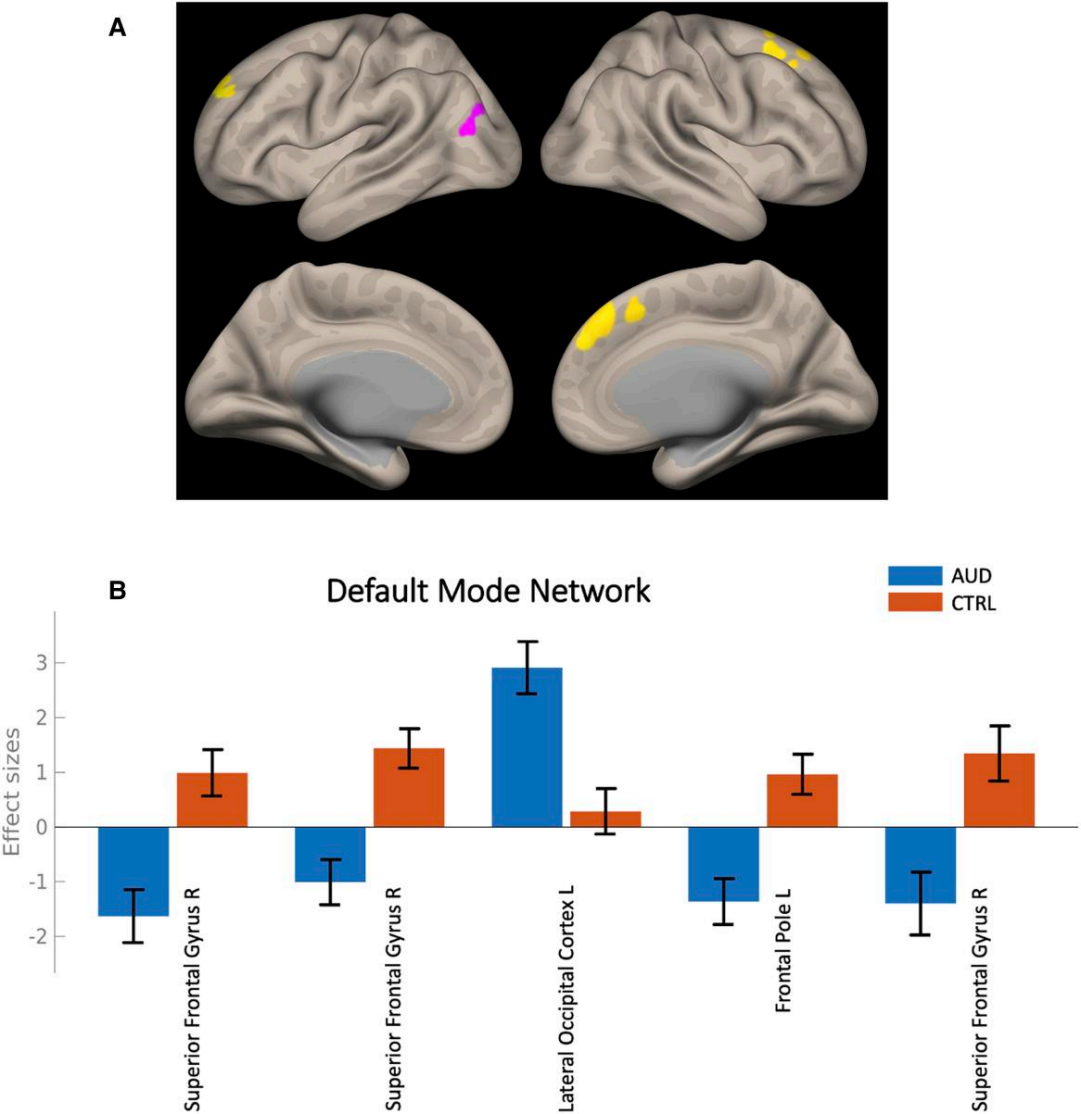
(**A**) The map represents regions with significant differences in functional connectivity between the AUD versus CTRL groups determined using GLM. In this analysis, the cluster-level FDR-corrected threshold was set at *P* < 0.05 based on Gaussian random field theory. Yellow signifies regions where net connectivity differences between AUD and CTRL were negative values, and Pink signifies regions where net connectivity differences between AUD and CTRL were positive values. (**B**) The bar graphs represent the average differences in Fisher-transformed correlation values between the AUD and CTRL groups, for clusters identified at the *p*-FDR threshold of *P* < 0.05. Error bars represent 90% confidence intervals for the estimates. Abbreviations: AUD, alcohol use disorder group; CTRL, control group; L, left hemisphere; R; right hemisphere.

Independent component analysis results are presented in Figs. 1, 2 and Table 3. Fig. 1 shows the spatial extent for 10 of the major independent components (i.e. functional network maps) identified using the probabilistic ICA, as described in the Methods section. The maps represent distinct intrinsically connected regions and replicate the well-established fc-fMRI networks that can be captured by one or more components. Group differences (AUD versus CTRL) in connectivity are summarized in Table 3. Significant differences were observed in the connectivity patterns of structures within five major networks: the default mode network (DMN), attention network, frontal-parietal network (FPN), frontal cortical network (FCN) and the cerebellar network. Fig. 2 shows group differences in functional connectivity patterns of the DMN. Overall, the AUD group had lower connectivity of the superior frontal gyrus and frontal lobes in the DMN.

**Table 3:** Comparison of connectivity differences in functional connectivity networks based on ICA

Network	X	Y	Z	Size	*p*_FWE_	*p*_FDR_	Region	Direction
DMN								
	8	42	40	161	0.000032	0.000032	Superior frontal gyrus (R)	AUD < CTRL
	16	20	48	110	0.000828	0.000415	Superior frontal gyrus (R)	AUD < CTRL
	−32	−80	26	74	0.011315	0.003804	Lateral occipital cortex, superior (L)	AUD > CTRL
	−16	54	34	57	0.044379	0.011380	Frontal pole (L)	AUD < CTRL
	16	30	42	42	0.159291	0.034799	Superior frontal gyrus (R)	AUD < CTRL
AN								
	−26	2	58	562	0.000000	0.000000	Superior frontal gyrus (L)	AUD < CTRL
	34	−78	−2	158	0.000072	0.000069	Lateral occipital cortex, inferior (R)	AUD > CTRL
	34	−8	66	84	0.007829	0.005013	Precentral gyrus (R)	AUD < CTRL
	44	−42	56	65	0.031615	0.011970	Superior parietal lobule (R)	AUD < CTRL
	−42	−42	50	63	0.036843	0.011970	Superior parietal lobule (R)	AUD < CTRL
	42	−62	−20	52	0.087200	0.024936	Temporal occipital fusiform (R)	AUD > CTRL
	10	44	40	124	0.000432	0.000819	Superior frontal gyrus (R)	AUD > CTRL
	2	−84	10	65	0.021385	0.029391	Supracalcarine cortex (R)	AUD > CTRL
FPN								
	54	−24	−14	84	0.004646	0.004978	Middle temporal gyrus, posterior (R)	AUD > CTRL
FCN								
	52	−14	44	94	0.003456	0.003103	Post-central/precentral gyrus (R)	AUD > CTRL
	−56	−6	38	81	0.008700	0.003916	Post-central/precentral gyrus (L)	AUD > CTRL
CN								
	28	16	−38	61	0.024493	0.020198	Temporal pole (R)	AUD > CTRL

*X*, *Y* and *Z* represent peak-voxel location within each cluster in the Montreal Neurological Institute (MNI) space; Size represents the size of the cluster; *p*_FWE_ represents cluster-size *P*-value corrected for family wise error; *p*_FDR_ represents cluster-size *P*-value corrected for false discovery rate. Abbreviations: DMN, default mode network; AN, attention network; FPN, frontoparietal network; FCN, frontal cortical network; CN, cerebellar network; (L), left hemisphere; (R), right hemisphere.

**Figure 3 Connectivity differences identified based on dynamic ICA.**
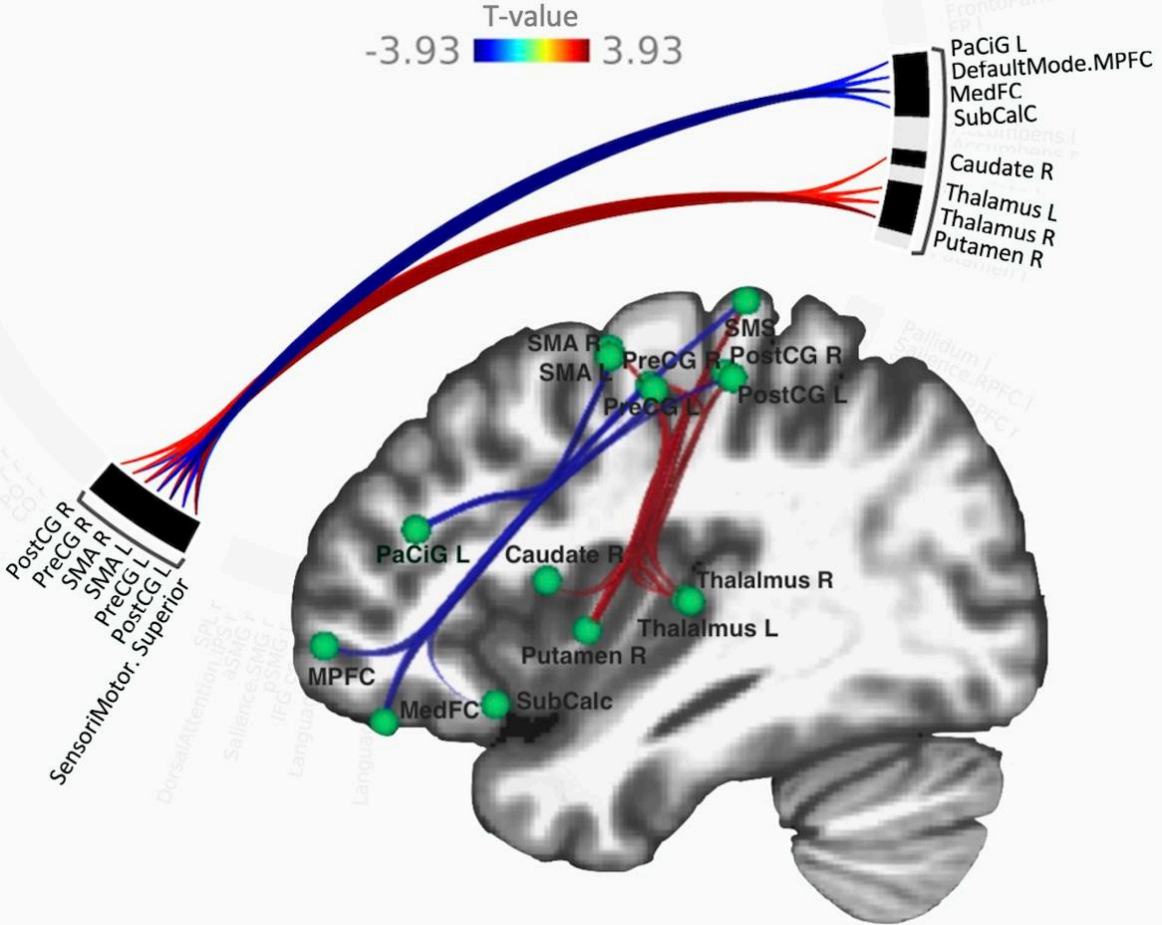
A cluster of regions was identified showing differences based on dynamic ICA between the AUD and CTRL groups using GLM where cluster-based inference was based on cluster-level *p*-FDR-corrected threshold of *P* < 0.05 and using a MVPA omnibus test. The uncorrected region to region connection threshold in this analysis was also set at *P* < 0.05. Blue (AUD > CTRL) and red (CTRL > AUD) indicate differences in dynamic functional connectivity. The colour bar indicates T-statistics values. Warmer (red-yellow) colours represent decreased dynamic FC in AUD (AUD < CTRL), colder (blue) colours represent increased dynamic FC strength in AUD (AUD > CTRL). Abbreviations: PreCG, precentral gyrus; PostCG, post-central gyrus; SMA, supplementary motor area; MPFC, medial prefrontal cortex; MedFC, medial frontal cortex; SubCalC, subcallosal cortex; PaCiG, paracingulate gyrus; L, left hemisphere; R, right hemisphere.

Dynamic ICA results are presented in Fig. 3. This analysis reveals circuits of connections with similar modulation in connectivity over time that explain most of the temporal variability in connectivity observed in the data. The results shown here represent a cluster compromising of 15 regions and 34 connections that showed a significant difference in dynamic functional connectivity between the AUD and CTRL (*F(2,50)* = 9.45, *p*_uncorrected_ = 0.0003, *p*_FDR_ = 0.021). The cluster of connections consisted of the following regions: subcortical reward regions (putamen, caudate and thalamus), cortical somatosensory regions (precentral gyrus, post-central gyrus, supplementary motor area and superior sensorimotor network) and frontal cortical regions (medial frontal cortex including medial prefrontal cortex of the DMN, subcallosal cortex; and the paracingulate gyrus). Additionally, in the cluster of regions identified using dynamic ICA, there was stronger functional connectivity in time between subcortical reward regions and somatosensory regions in the AUD group, and stronger functional connectivity in time between somatosensory regions and frontal cortical regions in the CTRL group.

Additional analyses revealed differences in the temporal variability of the BOLD timeseries between AUD and CTRL in a network consisting of only the basal ganglia and thalamus (*P* < 0.05), see Fig. 1. We established that connectivity strength between certain nodes/regions within the cluster identified through dynamic-ICA showed significant linear correlation with AUD-related measures as summarized in Table 4. Notably, there was a significant positive correlation between the putamen and somatosensory cortical regions and LOS.

**Table 4:** Correlation between AUD and connectivity strengthsindices

AUD measures	Connections	Correlation^a^
Length of sobriety	Putamen (R) and SMA (R)	0.479
Length of sobriety	Putamen (R) and PreCG (L)	0.432
Daily drinks	PreCG (L) and DefaultMode.MPFC	−0.463
Daily drinks	PreCG (L) and SubCalC	−0.514

^a^ Pearson correlation coefficient representing the strength and direction of linear relationships between an AUD-related measures and connectivity strength between regions in the cluster/network of regions identified in the dynamic ICA. All correlations were significant at *P < 0.05*. Abbreviations: SMA, supplementary motor area; PreCG, precentral gyrus; MPFC, medial prefrontal cortex; SubCalC, subcalcarine cortex.

## Discussion

Abnormalities of temporal dynamics of functional connectivity may interfere with global synchronization of the brain in numerous neurological and psychiatric disorders including AUD.^39^ In this study, we identified differences in brain functional connectivity patterns between individuals with a history of AUD and those with no history of alcohol misuse. We observed between-group differences in static intrinsic functional connectivity for default mode, attention, frontal-parietal, frontal cortical and cerebellar networks, as well as differences between groups in the dynamic intrinsic functional connectivity involving key regions in reward, sensorimotor and frontal cortical functional networks.

The ICA findings highlighted group differences in static intrinsic functional connectivity spanning multiple networks in the brain. Differences in DMN IC mainly involved the superior frontal gyrus and frontal pole. While the meaning of abnormal connectivity patterns is not completely understood, these results are consistent with well-documented reports of AUD-related brain damage and abnormalities that lead to a propensity for developing AUD, primarily affecting frontal cortical regions.^10,40,41^ Such damage can disrupt normal inhibitory functioning (e.g. inhibiting inappropriate responses^42^), or evaluative functions (e.g. evaluation of the experience of pain and modulation of pain^43^) involving the prefrontal cortex. Interestingly, our results showed group differences in connectivity between frontal cortical regions of the FCN with sensorimotor pericentral cortical regions, perhaps reflecting abnormalities in cortico-basal ganglia-thalamo-cortical loops underlying habitual ethanol seeking behaviour.^44^ Reductions in regional homogeneity (a measure reflective of temporal coherence between the BOLD timeseries of nearest neighbours of a voxel) also have been reported for the left post-central gyrus in association with alcohol dependence, as well as decreased functional connectivity between the left precentral region and the left cerebellum in individuals who relapse.^21^

We also observed abnormal DMN connectivity associated with AUD, consistent with patterns observed in substance use disorders.^45^ Substance abuse alters DMN connectivity affecting cognitive and emotional processing.^45^ Our results highlight reduced connectivity of frontal cortical regions (superior frontal gyrus and frontal pole) with other DMN regions in the AUD group. Differences also were observed in connectivity patterns with the frontal, parietal and occipital cortical regions for the attention network, perhaps reflecting attention deficits in the context of cognitive impairments in AUD.^46^ Attention deficits are widely reported in alcoholism and are possibly related to the sensitivity of frontal and parietal brain regions to chronic alcohol use,^47,48^ or associated with a predisposed vulnerability to alcoholism.^49^

Additionally, we observed connections that were stronger in the AUD group than in the CTRL group, which could be reflective of pre-existing risk factors, recovery, or compensatory mechanisms occurring with prolonged abstinence.^10,27^ For example, we observed increased connectivity of the right temporal pole with the cerebellum, a structure previously reported to show increased fMRI activity in AUD, and postulated to compensate for deficient frontal and parietal activity.^27^ In turn, increased connectivity with the cerebellum might help to maintain or improve cognitive abilities, such as inhibitory control functions.^50,51^ In non-abstinent alcoholics, task-based fMRI studies have demonstrated alcoholism-related hyperactivation (perhaps related to increased effort or compensatory effects required in the context of cerebral damage)^27^ in a variety of brain regions, including the bilateral dorsolateral prefrontal cortex while performing an emotional encoding task^52^ or a delayed-reward decision-making task,^53^ and right cerebellar and left frontal (language-control) regions during a verbal-working memory task.^54^

Our results further indicate a cluster of dynamically connected regions in which there is stronger dynamic connectivity between subcortical reward regions (including putamen) and cortical sensorimotor regions, and a lower connectivity between the frontal cortical regions (including medial prefrontal cortex) and sensorimotor regions in AUD. Several of these regions have been implicated in habit formation. Habit formation has been linked to drug-induced changes or adaptations in neural pathways that mediate the interactions between the functional brain networks that are implicated in addiction.^55,56^ Aberrant reward processing in the brain is an example of one such change that has been associated with development and maintenance of chronic alcohol use,^57^ craving,^58^ and risk of relapse.^59^ The reward network is involved in the anticipation of reward and regulation of related emotions.^60^ Interactions between sensory input (not direct) and reward pathways play an important role in goal-directed behaviour.^61^ The greater connectivity between subcortical regions of the reward and sensorimotor networks was countered by the reduced connectivity in AUD group between the same sensorimotor regions and the frontal cortical regions that also mediate reward and motivation. Of interest, one of the key areas implicated in our findings is the putamen, a key hub in the brain implicated in habit formation and the development of automatic behaviours.^62^

Functional connectivity facilitates integration of information across distinct specialized brain regions that ultimately enable various brain functions.^63^ Any impairment in functional connection between brain regions or a network of regions could interfere with execution of function(s). Our results particularly highlight lower dynamic functional connectivity between the thalamus and basal ganglia with sensorimotor cortical regions in AUD versus CTRL. We also found that there is an increase in connectivity between some of those regions with longer duration of sobriety (Table 4). We interpret all the above findings as suggestive of information integration impairments between reward and sensorimotor regions in AUD that seemingly improve with longer duration of sobriety. However, we also found higher temporal variability of the BOLD timeseries in AUD versus CTRL in an ICA network comprised of only the basal ganglia and thalamus. Higher regional or local BOLD signal temporal variability is most commonly thought to be reflective of greater functional integration,^64^ but within a network, higher variability of the BOLD signal temporal variability may be reflective of lower functional integration^65^ and/or compensatory mechanisms.^64,66^

We also found static functional connectivity in the AUD group to be predominantly affected in networks involving the frontal cortical regions, while at the same time, the temporal variability of functional connectivity was elevated within a network comprised of the basal ganglia and thalamus. Together, these findings are suggestive of increased irregularity in the cortico-basal ganglia-thalamo-cortical loops. It is likely that increased variability in the basal ganglia-thalamic connections may actually be modulating the synchrony between other cortical connections, particularly those involving regions in the frontal lobes. This is something that would not have been captured by measures of static functional connectivity alone, as static connectivity assumes that the functional connectivity remains a constant representation of the average functional connectivity between different regions during a resting period.^67^ Rather, as suggested by our findings, the AUD-related modulatory influences may be better characterized by dynamic functional connectivity. This finding also is consistent with multiple lines of evidence supporting a role for AUD-related abnormalities in the reward system.^55,68,69^ In the context of AUD, the modulatory network may be a predominantly subcortical network that disrupts the synchrony of the cortico-basal ganglia-thalamo-cortical loops by interfering with functioning of specific static cortical hubs.

Additionally, we observed that the AUD group showed stronger static functional connectivity between the sensorimotor cortex and the FCN, whereas the dynamic connectivity was lower between the same sensorimotor and frontal regions (including medial prefrontal cortex). These observations may be indicative of AUD-related deficits in processing and integration of sensory information,^70^ and the increased static connectivity may reflect a compensatory mechanism^27^ for reducing the connectivity instability over time.

It is important to note that while static and dynamic functional connectivity overlapped in identifying abnormalities in both frontal cortical and sensorimotor regions, there were aspects of the difference between AUD and CTRL groups that were captured by measures of dynamic connectivity but not by static connectivity. Most notably, our study captured the temporal properties of functional connectivity and showed AUD-related deficits of dynamic connections involving thalamus and basal ganglia. Disruption of these connections can interfere with various aspects of motivation, cognition and motor control.^71^

### Limitations

The present study was a cross-sectional design; therefore, it is unknown whether the functional connectivity abnormalities in the AUD group are a result of heavy drinking or a predisposition toward AUD. Although the average LOS within the AUD group was long (5.2 +/−7.2 years), we chose a wide range of abstinence by design to generalize our findings to the larger population of AUD men and women in our catchment area. However, we performed additional analyses that clearly indicated a correlation between LOS and connectivity patterns that were identified to be different between the AUD and CTRL groups.

Additionally, because our sample of AUD participants was successful in maintaining abstinence, the results may not generalize to AUD individuals who have more difficulty with relapse prevention. The present study also did not examine the causal relationship of abstinence and connectivity patterns, and thus, less is known about whether observed patterns of network connectivity play a role in maintaining abstinence from alcohol, or that continued abstinence results in changes in connectivity. Moreover, the present study did not examine the trajectory of abstinence on network connectivity, though previous literature of short- and long-term abstinence suggests that continued sobriety leads to stronger connectivity between certain brain networks as a function of compensation or recovery effects.^72^

Finally, because this study was cross-sectional and not longitudinal, caution is recommended in interpreting the findings in terms of their implications for recovery and changes that occur over time. Importantly, while AUD individuals reported varying LOS, it is not necessarily a proxy for chronicity or reflective of the severity of AUD. In other words, taken together, the LOS correlations reported herein, are not necessarily indicative of changes in connectivity patterns due to recovery.

## Conclusions

Results from this study described abnormalities in intrinsic functional connectivity patterns associated with AUD. Our findings highlight alterations in functional connectivity spanning multiple networks in the brain mainly involving frontal cortical, sensorimotor and subcortical reward network regions. Since there was a wide range of abstinence in the AUD group, including individuals with long periods of sobriety, the observed patterns of abnormal connectivity may reflect persistent AUD-related network abnormalities, compensatory recovery-related processes whereby additional neural resources are recruited to achieve normal levels of performance,^27^ or a predisposition toward developing AUD.

## Abbreviations

AUD =: alcohol use disorder
AUDIT =: Alcohol Use Disorders Identification Test
BOLD =: blood oxygenation level dependent
CTRL =: control group
DSM-IV =: diagnostic and statistical manual of mental disorders, fourth edition
DMN =: default mode network
IC =: intrinsic connectivity
ICA =: independent component analysis
FCN =: frontal cortical network
FDR =: false discovery rate
fMRI =: functional magnetic resonance imaging
fc-fMRI =: functional connectivity fMRI
rs-fMRI =: resting-state fMRI
FPN =: frontal-parietal network
FWHM =: full-width-at-half-maximum
gPPI =: generalized psycho-physiological interaction
LOS =: length of sobriety
MVPA =: multi-variate pattern analysis
ROI =: region of interest

## References

[1] SAMHSA . 2019 National Survey on Drug Use and Health—SAMHSA. 2019. Accessed 8 January 2022. http://www.samhsa.gov/data/sites/default/files/reports/rpt29394/NSDUHDetailedTabs2019/NSDUHDetTabsSect5pe2019.htm

[2] Rourke SB . The neurobehavioral correlates of alcoholism. In: Grant I, ed. Neuropsychological assessment of neuropsychiatric and neuromedical disorders. 3rd edn. Oxford University Press; 2009:398–454.

[3] Crews FT , Buckley T, Dodd PR, et al Alcoholic neurobiology: Changes in dependence and recovery. Alcohol Clin Exp Res. 2005;29(8):1504–1513. 16156047 10.1097/01.alc.0000175013.50644.61

[4] Reilly MT , Noronha A, Warren K. Perspectives on the neuroscience of alcohol from the National Institute on Alcohol Abuse and Alcoholism. Handb Clin Neurol. 2014; 125:15–29. 25307566 10.1016/B978-0-444-62619-6.00002-1

[5] Nutt D , Hayes A, Fonville L, et al Alcohol and the brain. Nutrients. 2021;13(11):3938. 34836193 10.3390/nu13113938 PMC8625009

[6] Zahr NM , Pfefferbaum A. Alcohol's effects on the brain: Neuroimaging results in humans and animal models. Alcohol Res. 2017;38(2):183–206. 28988573 10.35946/arcr.v38.2.04 PMC5513685

[7] Squeglia LM , Jacobus J, Tapert SF. The effect of alcohol use on human adolescent brain structures and systems. Handb Clin Neurol. 2014;125:501–510. 25307592 10.1016/B978-0-444-62619-6.00028-8 PMC4321715

[8] Hillmer AT , Mason GF, Fucito LM, O'Malley SS, Cosgrove KP. How imaging glutamate, γ-aminobutyric acid, and dopamine can inform the clinical treatment of alcohol dependence and withdrawal. Alcohol Clin Exp Res. 2015;39(12):2268–2282. 26510169 10.1111/acer.12893 PMC4712074

[9] Yang X , Tian F, Zhang H, et al Cortical and subcortical gray matter shrinkage in alcohol-use disorders: A voxel-based meta-analysis. Neurosci Biobehav Rev. 2016;66:92–103. 27108216 10.1016/j.neubiorev.2016.03.034

[10] Oscar-Berman M , Valmas MM, Sawyer KS, Ruiz SM, Luhar RB, Gravitz ZR. Profiles of impaired, spared, and recovered neuropsychologic processes in alcoholism. Handb Clin Neurol. 2014;125:183–210. 25307576 10.1016/B978-0-444-62619-6.00012-4 PMC4515358

[11] Oscar-Berman M , Ruiz SM, Marinkovic K, Valmas MM, Harris GJ, Sawyer KS. Brain responsivity to emotional faces differs in men and women with and without a history of alcohol use disorder. PLoS ONE. 2021;16(6):e0248831. 10.1371/journal.pone.0248831 PMC8189468 34106934

[12] Sawyer KS , Maleki N, Urban T, et al Alcoholism gender differences in brain responsivity to emotional stimuli. Elife. 2019;8:e41723. 31038125 10.7554/eLife.41723 PMC6491039

[13] Fritz M , Klawonn AM, Zahr NM. Neuroimaging in alcohol use disorder: From mouse to man. J Neurosci Res. 2022;100(5):1140–1158. 31006907 10.1002/jnr.24423 PMC6810809

[14] Sawyer KS , Maleki N, Papadimitriou G, Makris N, Oscar-Berman M, Harris GJ. Cerebral white matter sex dimorphism in alcoholism: A diffusion tensor imaging study. Neuropsychopharmacology. 2018;43(9):1876–1883. 29795404 10.1038/s41386-018-0089-6 PMC6046037

[15] Sawyer KS , Oscar-Berman M, Barthelemy OJ, Papadimitriou GM, Harris GJ, Makris N. Gender dimorphism of brain reward system volumes in alcoholism. Psychiatry Res Neuroimaging. 2017;263:15–25. 28285206 10.1016/j.pscychresns.2017.03.001 PMC5415444

[16] Sawyer KS , Oscar-Berman M, Mosher Ruiz S, et al Associations between cerebellar subregional morphometry and alcoholism history in men and women. Alcohol Clin Exp Res. 2016;40(6):1262–1272. 27130832 10.1111/acer.13074 PMC4889497

[17] Valmas MM , Mosher Ruiz S, Gansler DA, Sawyer KS, Oscar-Berman M. Social cognition deficits and associations with drinking history in alcoholic men and women. Alcohol Clin Exp Res. 2014;38(12):2998–3007. 25581654 10.1111/acer.12566 PMC4293081

[18] Ruiz SM , Oscar-Berman M, Sawyer KS, Valmas MM, Urban T, Harris GJ. Drinking history associations with regional white matter volumes in alcoholic men and women. Alcohol Clin Exp Res. 2013;37(1):110–122. 22725728 10.1111/j.1530-0277.2012.01862.x PMC3459287

[19] Kwong KK , Belliveau JW, Chesler DA, et al Dynamic magnetic resonance imaging of human brain activity during primary sensory stimulation. Proc Natl Acad Sci USA. 1992;89(12):5675–5679. 1608978 10.1073/pnas.89.12.5675 PMC49355

[20] Finn ES . Is it time to put rest to rest? Trends Cogn Sci. 2021;25(12):1021–1032. 34625348 10.1016/j.tics.2021.09.005 PMC8585722

[21] Deng R , Yang X, Meng YJ, et al Data-driven study on resting-state functional magnetic resonance imaging during early abstinence of alcohol dependence in male patients and its predictive value for relapse. BMC Psychiatry. 2022;22(1):143. 35193538 10.1186/s12888-022-03782-w PMC8862392

[22] Gratton C , Laumann TO, Nielsen AN, et al Functional brain networks are dominated by stable group and individual factors. Not cognitive or daily variation. Neuron. 2018;98(2):439–452.e5. 29673485 10.1016/j.neuron.2018.03.035 PMC5912345

[23] Zhu X , Cortes CR, Mathur K, Tomasi D, Momenan R. Model-free functional connectivity and impulsivity correlates of alcohol dependence: A resting-state study. Addict Biol. 2017;22(1):206–217. 26040546 10.1111/adb.12272 PMC4669235

[24] Chanraud S , Pitel AL, Pfefferbaum A, Sullivan EV. Disruption of functional connectivity of the default-mode network in alcoholism. Cereb Cortex. 2011;21(10):2272–2281. 21368086 10.1093/cercor/bhq297 PMC3169657

[25] Canessa N , Basso G, Carne I, Poggi P, Gianelli C. Increased decision latency in alcohol use disorder reflects altered resting-state synchrony in the anterior salience network. Sci Rep. 2021;11(1):19581. 34599268 10.1038/s41598-021-99211-1 PMC8486863

[26] Müller-Oehring EM , Jung YC, Sullivan EV, Hawkes WC, Pfefferbaum A, Schulte T. Midbrain-driven emotion and reward processing in alcoholism. Neuropsychopharmacology. 2013;38(10):1844–1853. 23615665 10.1038/npp.2013.102 PMC3746685

[27] Oscar-Berman M , Marinković K. Alcohol: Effects on neurobehavioral functions and the brain. Neuropsychol Rev. 2007;17(3):239–257. 17874302 10.1007/s11065-007-9038-6 PMC4040959

[28] Saunders JB , Aasland OG, Babor TF, de la Fuente JR, Grant M. Development of the Alcohol Use Disorders identification test (AUDIT): WHO collaborative project on early detection of persons with harmful alcohol consumption–II. Addiction. 1993;88(6):791–804. 8329970 10.1111/j.1360-0443.1993.tb02093.x

[29] Robins LN , Cottler LB, Bucholz KK, Compton WM, North CS, Rourke KM. Diagnostic interview schedule for the DSM-IV (DIS-IV). Washington University School of Medicine; 2000.

[30] American Psychiatric Association . Diagnostic and statistical manual of mental disorders. 4th edn. American Psychiatric Association Publishing; 1994.

[31] Zuckerman M , Lubin B. Multiple affect adjective check list. Educational and Industrial Testing Services; 1965.

[32] Behzadi Y , Restom K, Liau J, Liu TT. A component based noise correction method (CompCor) for BOLD and perfusion based fMRI. Neuroimage. 2007;37(1):90–101. 17560126 10.1016/j.neuroimage.2007.04.042 PMC2214855

[33] Whitfield-Gabrieli S , Nieto-Castanon A. Conn: A functional connectivity toolbox for correlated and anticorrelated brain networks. Brain Connect. 2012;2(3):125–141. 22642651 10.1089/brain.2012.0073

[34] Goldstein JM , Seidman LJ, Makris N, et al Hypothalamic abnormalities in schizophrenia: Sex effects and genetic vulnerability. Biol Psychiatry. 2007;61(8):935–945. 17046727 10.1016/j.biopsych.2006.06.027

[35] Desikan RS , Ségonne F, Fischl B, et al An automated labeling system for subdividing the human cerebral cortex on MRI scans into gyral based regions of interest. Neuroimage. 2006;31(3):968–980. 16530430 10.1016/j.neuroimage.2006.01.021

[36] Makris N , Goldstein JM, Kennedy D, et al Decreased volume of left and total anterior insular lobule in schizophrenia. Schizophr Res. 2006;83(2-3):155–171. 16448806 10.1016/j.schres.2005.11.020

[37] Frazier JA , Chiu S, Breeze JL, et al Structural brain magnetic resonance imaging of limbic and thalamic volumes in pediatric bipolar disorder. Am J Psychiatry. 2005;162(7):1256–1265. 15994707 10.1176/appi.ajp.162.7.1256

[38] Tzourio-Mazoyer N , Landeau B, Papathanassiou D, et al Automated anatomical labeling of activations in SPM using a macroscopic anatomical parcellation of the MNI MRI single-subject brain. Neuroimage. 2002;15(1):273–289. 11771995 10.1006/nimg.2001.0978

[39] Greene DJ , Marek S, Gordon EM, et al Integrative and network-specific connectivity of the basal ganglia and thalamus defined in individuals. Neuron. 2020;105(4):742–758.e6. 31836321 10.1016/j.neuron.2019.11.012 PMC7035165

[40] Mosher Ruiz S , Oscar-Berman M, Kemppainen MI, Valmas MM, Sawyer KS. Associations between personality and drinking motives among abstinent adult alcoholic men and women. Alcohol Alcohol. 2017;52(4):496–505. 28379312 10.1093/alcalc/agx016 PMC6322439

[41] Oscar-Berman M , Valmas MM, Sawyer KS, et al Frontal brain dysfunction in alcoholism with and without antisocial personality disorder. Neuropsychiatr Dis Treat. 2009;5:309–326. 19557141 10.2147/ndt.s4882 PMC2699656

[42] Sakagami M , Pan X, Uttl B. Behavioral inhibition and prefrontal cortex in decision-making. Neural Netw. 2006;19(8):1255–1265. 16952442 10.1016/j.neunet.2006.05.040

[43] Qin Z , Su J, He XW, et al Disrupted functional connectivity between sub-regions in the sensorimotor areas and cortex in migraine without aura. J Headache Pain. 2020;21(1):47. 32375638 10.1186/s10194-020-01118-1 PMC7203097

[44] Lovinger DM , Alvarez VA. Alcohol and basal ganglia circuitry: Animal models. Neuropharmacology. 2017;122:46–55. 28341206 10.1016/j.neuropharm.2017.03.023 PMC5479739

[45] Zhang R , Volkow ND. Brain default-mode network dysfunction in addiction. Neuroimage. 2019;200:313–331. 31229660 10.1016/j.neuroimage.2019.06.036

[46] Stavro K , Pelletier J, Potvin S. Widespread and sustained cognitive deficits in alcoholism: A meta-analysis. Addict Biol. 2013;18(2):203–213. 22264351 10.1111/j.1369-1600.2011.00418.x

[47] Ratti MT , Bo P, Giardini A, Soragna D. Chronic alcoholism and the frontal lobe: Which executive functions are imparied? Acta Neurol Scand. 2002;105(4):276–281. 11939939 10.1034/j.1600-0404.2002.0o315.x

[48] Oscar-Berman M , Kirkley SM, Gansler DA, Couture A. Comparisons of Korsakoff and non-Korsakoff alcoholics on neuropsychological tests of prefrontal brain functioning. Alcohol Clin Exp Res. 2004;28(4):667–675. 15100620 10.1097/01.alc.0000122761.09179.b9 PMC4074361

[49] Peterson JB , Pihl RO. Information processing, neuropsychological function, and the inherited predisposition to alcoholism. Neuropsychol Rev. 1990;1(4):343–369. 2152535 10.1007/BF01109029

[50] Qiu Z , Wang J. Altered neural activities during response inhibition in adults with addiction: A voxel-wise meta-analysis. Psychol Med. 2021;51(3):387–399. 33612127 10.1017/S0033291721000362

[51] Sullivan EV , Pfefferbaum A. Neurocircuitry in alcoholism: A substrate of disruption and repair. Psychopharmacology (Berl). 2005;180(4):583–594. 15834536 10.1007/s00213-005-2267-6

[52] Marinkovic K , Oscar-Berman M, Urban T, et al Alcoholism and dampened temporal limbic activation to emotional faces. Alcohol Clin Exp Res. 2009;33(11):1880–1892. 19673745 10.1111/j.1530-0277.2009.01026.x PMC3543694

[53] Amlung M , Sweet LH, Acker J, Brown CL, MacKillop J. Dissociable brain signatures of choice conflict and immediate reward preferences in alcohol use disorders. Addict Biol. 2014;19(4):743–753. 23231650 10.1111/adb.12017 PMC3871988

[54] Desmond JE , Chen SH, DeRosa E, Pryor MR, Pfefferbaum A, Sullivan EV. Increased frontocerebellar activation in alcoholics during verbal working memory: An fMRI study. Neuroimage. 2003;19(4):1510–1520. 12948707 10.1016/s1053-8119(03)00102-2

[55] Koob GF , Volkow ND. Neurobiology of addiction: A neurocircuitry analysis. Lancet Psychiatry. 2016;3(8):760–773. 27475769 10.1016/S2215-0366(16)00104-8 PMC6135092

[56] Volkow ND , Morales M. The brain on drugs: From reward to addiction. Cell. 2015;162(4):712–725. 26276628 10.1016/j.cell.2015.07.046

[57] Setiawan E , Pihl RO, Dagher A, et al Differential striatal dopamine responses following oral alcohol in individuals at varying risk for dependence. Alcohol Clin Exp Res. 2014;38(1):126–134. 23919483 10.1111/acer.12218

[58] Fox HC , Bergquist KL, Hong KI, Sinha R. Stress-induced and alcohol cue-induced craving in recently abstinent alcohol-dependent individuals. Alcohol Clin Exp Res. 2007;31(3):395–403. 17295723 10.1111/j.1530-0277.2006.00320.x

[59] Heinz A , Beck A, Grüsser SM, Grace AA, Wrase J. Identifying the neural circuitry of alcohol craving and relapse vulnerability. Addict Biol. 2009;14(1):108–118. 18855799 10.1111/j.1369-1600.2008.00136.x PMC2879014

[60] Liu J , Claus ED, Calhoun VD, Hutchison KE. Brain regions affected by impaired control modulate responses to alcohol and smoking cues. J Stud Alcohol Drugs. 2014;75(5):808–816. 25208199 10.15288/jsad.2014.75.808 PMC4161701

[61] Haber SN , Knutson B. The reward circuit: Linking primate anatomy and human imaging. Neuropsychopharmacology. 2010;35(1):4–26. 19812543 10.1038/npp.2009.129 PMC3055449

[62] Balleine BW , O'Doherty JP. Human and rodent homologies in action control: Corticostriatal determinants of goal-directed and habitual action. Neuropsychopharmacology. 2010;35(1):48–69. 19776734 10.1038/npp.2009.131 PMC3055420

[63] Friston KJ . Functional and effective connectivity: A review. Brain Connect. 2011;1(1):13–36. 22432952 10.1089/brain.2011.0008

[64] Nomi JS , Bolt TS, Ezie CEC, Uddin LQ, Heller AS. Moment-to-moment BOLD signal variability reflects regional changes in neural flexibility across the lifespan. J Neurosci. 2017;37(22):5539–5548. 28473644 10.1523/JNEUROSCI.3408-16.2017 PMC5452342

[65] Yin D , Kaiser M. Understanding neural flexibility from a multifaceted definition. Neuroimage. 2021;235:118027. 33836274 10.1016/j.neuroimage.2021.118027

[66] Garrett DD , Epp SM, Perry A, Lindenberger U. Local temporal variability reflects functional integration in the human brain. Neuroimage. 2018;183:776–787. 30149140 10.1016/j.neuroimage.2018.08.019

[67] Calhoun VD , Miller R, Pearlson G, Adalı T. The chronnectome: Time-varying connectivity networks as the next frontier in fMRI data discovery. Neuron. 2014;84(2):262–274. 25374354 10.1016/j.neuron.2014.10.015 PMC4372723

[68] Bowirrat A , Oscar-Berman M. Relationship between dopaminergic neurotransmission, alcoholism, and reward deficiency syndrome. Am J Med Genet B Neuropsychiatr Genet. 2005;132B(1):29–37. 15457501 10.1002/ajmg.b.30080

[69] Koob GF , Rassnick S, Heinrichs S, Weiss F. Alcohol, the reward system and dependence. EXS. 1994;71:103–114. 7913351 10.1007/978-3-0348-7330-7_11

[70] Groenewegen HJ , Uylings HB. The prefrontal cortex and the integration of sensory, limbic and autonomic information. Prog Brain Res. 2000;126:3–28. 11105636 10.1016/S0079-6123(00)26003-2

[71] Haber SN , Calzavara R. The cortico-basal ganglia integrative network: The role of the thalamus. Brain Res Bull. 2009;78(2-3):69–74. 18950692 10.1016/j.brainresbull.2008.09.013 PMC4459637

[72] Camchong J , Stenger VA, Fein G. Resting state synchrony in long-term abstinent alcoholics with versus without comorbid drug dependence. Drug Alcohol Depend. 2013;131(1-2):56–65. 23639390 10.1016/j.drugalcdep.2013.04.002 PMC3759679

